# Are Cosmetics Used in Developing Countries Safe? Use and Dermal Irritation of Body Care Products in Jimma Town, Southwestern Ethiopia

**DOI:** 10.1155/2012/204830

**Published:** 2012-11-01

**Authors:** Wayessa Amasa, Dante Santiago, Seblework Mekonen, Argaw Ambelu

**Affiliations:** ^1^Department of Eco-Bology, Addis Ababa Science and Technology University, P.O. Box 16417, Addis Ababa, Ethiopia; ^2^Department of Environmental Health Sciences and Technology, College of Public Health and Medical Sciences, Jimma University, P.O. Box 378, Jimma, Ethiopia; ^3^Department of Environmental Health Sciences and Technology, College of Public Health and Medical Sciences, Jimma University, P.O. Box 807, Jimma, Ethiopia

## Abstract

*Background*. Rabbit skin model was used to test skin irritation of the most commonly used cosmetic products in Jimma town, southwestern Ethiopia. The most commonly used cosmetics were Dove, Glysolid, College, Top Society, Fair and Lovely, Nivea, Lux, Magic fruit world, Solea, Body talk, Kris, Holly, Victoria, and Sweet Heart. *Methods*. Intact and abraded rabbit skins were tested for erythema and edema under shade and under sun exposure. Draize Primary Irritation Index (PII) was used to calculate skin irritation of each cosmetic. Cosmetic ingredients were analyzed from the labels. *Results and Discussion*. Only Dove cream caused no skin irritation except for an abraded skin under sun exposure for five consecutive days. It has been identified that application of cosmetics on abraded skin under sunny condition worsens the irritation. Cosmetic labels revealed that most ingredients used in all products were those restricted chemicals due to their adverse health effects. *Conclusion*. This study has concluded that use of cosmetics under sunshine and also on abraded skin increases skin irritation. Hence, those users who have abraded skin are advised not to apply those cosmetics on continuous basis specifically under sun exposure.

## 1. Introduction

Cosmetics refer to all of the products used to care for and clean the human skin and make it more beautiful. The intentions of using cosmetic products is to maintain the body in a good condition, protect it from the effects of the environment and aging processes, change the appearance, and make the body smell nicer. Cosmetic products are widely used by every socioeconomic class of human beings to cleanse, perfume, protect, and change the appearance of skin [1].

Public observations and reviewing of the relevant literatures indicated that most of the cosmetic users were not seriously concerned about the effect of usage of products to their skin and focus on the short term result of skin appearance rather than the long-term effects to the whole body. Generally, consumers assume that cosmetic products are safer and pose no risk to the human health [2]. Some consumers did not read the label to identify the ingredients and other useful information of the cosmetic products before they decide to use them. The cosmetics sector grows tremendously, driven by demands from consumers but some users are not very concerned about the implications of cosmetics to their healthy body such as skin and physical outlook [2, 3].

In Ethiopia, cosmetics do not need marketing authorization unlike that of medicinal products which can only be marketed if marketing authorization is granted. DACA do not spend little or no time to protect the public against the harmful effects emanating from these products as it is true also in many other countries [4–7]. It is the companies' responsibility to check the safety of the cosmetic products and these firms are business oriented and give little or no attention to the cosmetics safety because of many reasons. Some of them are the requirement to change test methods, formulations, packaging, and advertising could increase costs for the sector [8].

Therefore there could be inadequacies and inconsistencies in container label disclosure by manufacturers [9]. These things create a gap in the event of defective product release in to the market [10]. Labeling is required for all cosmetic products and safety information must appear on cosmetic containers or packaging. According to Health Science Authority, labels on cosmetics should contain the function of the cosmetic product, instruction for use, list of all ingredients, country of manufacture, content (weight/volume), batch number, and manufacturing and expiry dates [11]. Cosmetics and personal-care products may contain ingredients whose safety is unclear or which are known to pose health risks. Adverse reactions to cosmetics are the commonest single reason for hospital referrals with allergic contact dermatitis [12, 13].

Most chemicals are added to cosmetic product in the form of preservatives and fragrances to increase the shelf life of the product and to have a good odor and appearance to the users [14]. These additive chemicals are the most common cause of skin problems such as skin irritation, photo toxicity, contact allergy, and other dermatological problems [15–17]. Documents indicate that in the previous times extracts of natural materials were used but currently synthetic ingredients are often used in the cosmetic products [18]. Some of these synthetic additives could be dangerous for the consumers' health. Estrogenic chemicals of body care cosmetics to the underarm and breast area are being investigated as a possible cause of breast cancer [19]. Literatures indicate that the concentration of lead in the candy lipstick cosmetic product is 2.8 times higher than the American FDA recommended concentration of lead [20]. 

Another problem associated with cosmetic use is the clarity of the labels on the cosmetic containers. The Tanzanian Food and Drug Authority have found that every container of any product shall be affixed with a label bearing legible and indelible letters in Kiswahili or Kiswahili and English: name of a cosmetic; form of a cosmetic; intended use; instructions on use of the cosmetic; net content given by weight or volume, in metric system; name and address of the manufacturer, including country of origin; dates of manufacture and expiry of a cosmetic in clear terms; list of ingredients in alphabetical order; batch or lot number; precautions and warnings; storage conditions where applicable [21]. Studies conducted somewhere else by the Environmental Defense in Canada have identified, using laboratory analysis, that heavy metals such as lead, arsenic, and antimony were found as ingredients which were not listed on the product label [22]. A similar study done has identified high levels of mercury in cream cosmetics [23]. Endocrine-disrupting chemicals were also identified in cosmetic products [24]. A study done in Nigeria also found an elevated level of lead in facial talcum powders and other metals in personal care products [25, 26].

A number of chemicals are usually added to cosmetics product as preservatives and fragrances. Many of them are toxic and prohibited from usage as ingredients because they can cause cancer, mutation, reproductive toxicity, and endocrine disruption. Preservatives added to skin cream products have been found above the maximum allowed concentration and do not comply with European Cosmetics Directives [27].

Cosmetics used for body care should be evaluated for irritancy potential to human skin of any chemical or formulation is a necessity. The most commonly used test is the rabbit skin irritation test as described in the OECD test guideline 404 and in the European Chemicals Bureau [28–30].

The nonmedicated cosmetics in Ethiopia are used without passing through structured safety evaluation and laboratory assessments. This might possibly lead to the presence and use of unsafe cosmetic products which could pose public health risk to the consumers. Hence, this study has been conducted to identify the safety of cosmetic products commonly used in urban communities considering Jimma as the case in point. This study helps the Federal Government of Ethiopia to formulate rules over the cosmetic products to protect the public safety and aware consumers. 

Therefore the main aim of this study was to assess the skin irritation of cosmetics most commonly used in Jimma town using rabbit as a test animal whose result can be directly predicted for human skin irritation for both erythema and edema.

## 2. Materials and Methods

### 2.1. Study Area and Period

The study was conducted in Jimma town southwest Ethiopia from April to June, 2011. According to the Central Statistics Agency the current estimated population of the town is about 650,000 [32]. Jimma has many shops in which cosmetics could be sold. According to the town Trade and Industry office, there are above 350 shops in which cosmetics are being sold. However, the popular shops in which majority of the consumers could buy cosmetics are about 15. For the purpose of this study, shops keepers from these shops were interviewed to identify the most commonly used cosmetic products. In addition, one working day (Saturday) onsite observation was done at each shop to verify consumer preferences to cosmetics and interview them for labeling and content of the product. Saturday was preferred because it is major shopping day in Ethiopia and therefore many consumers could be surveyed. For comparative purpose, the Ethiopian-made products, Kris (*Kri*) and Victoria (*Vic*) lotions, and one face make-up called Sweet Heart (*Swe*) were selected to be included in the study.

### 2.2. Test Rabbit

Six New Zealand white rabbits were bought from Ethiopian Health and Nutrition Research Institute in Addis Ababa. The rabbits were acclimatized to the laboratory environment prior to the experiment for a period of five days to normalize the animal's physiological status during transportation and adopt them to the environment. Each rabbit was given a number unique within the study written with a black indelible marker pen on the inner surface of the ear and on the cage label. The animals were individually housed in cages and kept at ambient temperature and relative humidity. 

### 2.3. Dermal Irritation Test Procedure

The selected cosmetics were tested for *skin irritation* using rabbits as test animals. Rabbit is a suitable model for this test of study since the results can be of value in predicting the likely skin irritancy potential of the test material to human [33].

One day before the test, six quadrants, 2.5 cm × 2.5 cm in dimension of each rabbit skin was clipped free of fur from the dorsal and flank area using shaving machines on five places for the selected cosmetics and one for the untreated control which was treated with distilled water. Only animals with healthy intact epidermis by gross observation were used for the study, and three rabbits were used per test.

#### 2.3.1. Toxicity Test on Intact Skin

A quantity of 0.5 g test material, moistened with 0.5 mL of physiological saline, was put onto a piece of cotton gauze patch and placed in position on the assigned quadrant on shorn skin. 0.5 mL physiological saline was mixed with the test material so as to make the product moistened and to be attached well to the rabbit skin. The patches were secured in position with a strip of surgical adhesive tape. To prevent the rabbit removing the patches, the trunk of each rabbit was wrapped in a corset, and the animals were kept in individual cages for the duration of the exposure period. Four hours after application, the corset and patches were removed from each animal and any residual test material, gently swabbed away with cotton wool soaked in distilled water. The test quadrants were examined for evidence of primary irritation both erythema (redness) and edema (swelling) at 24, 48, and 72 hours after removal of the cosmetic material. The standard procedure of the United States Testing Company, OECD, and European Chemicals Bureau for the Cosmetic Product uses similar procedures as it has been initially described by Draize and his colleagues in 1944 [31, 34].  Table 1 indicates erythema and edema scale based on United States Testing Company (USTC) scale.

#### 2.3.2. Toxicity Test on Abraded Skin

This test simulated the condition where the skin bears wound such as pimples and scratches. The same procedure as for the intact skin was used except that the shaved skin was rubbed with a fine abrasive paper five times.

#### 2.3.3. Daily Applications on Sun-Exposed Intact Skin

The intention of this test was to determine the effect of sun light on cosmetic products whether the sun light can activate the chemical species present in the products and cause irritation effect on the skin. This test is similar to Test 1 except that the cosmetics were applied on the bare rabbit skin for five consecutive days, and the rabbits were exposed to the sun or kept under shade for four hour per day. 

### 2.4. Determination of Primary Irritation Index

The duration of observation was three days for single topical application on intact and abraded skin but up to five days for repeated application. The primary irritation index (PII) for each treatment was calculated using the following formula:

(1) $$\begin{array}{c} \text{PII}=\frac{\Sigma \;\;\text{erythema grade at 24, 48 and 72}\;\text{hr + }\Sigma \;\;\text{edema grade at 24, 48 and 72 hr}}{\text{total number of observations}}. \end{array}$$

### 2.5. Interpretation of Results

The scores for erythema and edema at 24, 48, and 72 hours readings were totaled for the three test rabbits after the patch is removed and then divided by 18 to obtain the primary irritation index of the test material. The denominator 18 represents the number of rabbits used (three) multiplied by two types of observations (erythema and edema) multiplied by the three observation times (24, 48, and 72 hr), and the result was interpreted against the skin irritation category. According to Draize (1959), if the calculated PII is 0–0.4, 0.5–1.9, 2.0–4.9, and 5.0–8.0, the irritation category is, respectively, negligible, slight irritation, moderate irritation, and severe irritation [35].

## 3. Results

### 3.1. Cosmetics Survey

The cosmetic products identified in the survey as the most commonly bought by 433 consumers are given in Table 2. Top five cream cosmetic products most commonly used in Jimma town were Dove (*Dov*), Glysolid (*Gly*), College (*Col*), Top Society (*Top*), and Fair & Lovely (*Fai*) while the top five hand and body lotions were Body Talk (*Bod*), Nivea (*Niv*), Solea (*Sol*), Lux (*Lux*), and Magic Fruit World (*Mag*).

Consumers were asked whether they read the label on the container while buying the cosmetic product from the source (wholesalers) and nearly all of them (99.2%) do not pay attention to what is written on the labels of cosmetic products. However, about 98% of the consumers interviewed were aware and curious about the expiry date but other label information such as ingredients from which the product is made was not considered. Many consumers (78%) do not like to buy the Chinese, Indonesian, Indian, and Ethiopian products because they were not sure about the quality of the product and might not be safe to human health. In addition, most consumers (81%) do not trust products imported from china as well.

We have identified that the main criteria that the shopkeepers were bringing the cosmetic products to sale were only based on customer preferences and the shelf life marked on the label.

### 3.2. Product Ingredients

Based on the investigation made on the product labels, the different ingredients were identified. Most of the products those are commonly sold in Jimma town cosmetic shops contain prohibited ingredients due to their possible health effects. The ingredients were parabens, linalool, limonene, disodium EDTA, sodium lauryl sulphate, and triethanolamine (Table 5). 

No ingredient label was found on *Swe* and *Vic*. For some of the products, such as *Col*, *Niv*, *Mag,* and *Kri*, no expiry dates were mentioned.

### 3.3. Rabbit Model Experimental Result

#### 3.3.1. Single Application of Creams and Lotions on Intact and Abraded Rabbit Skin

After a single application on intact and abraded rabbit skin, Dov did not show any skin edema or erythema as the calculated PII score was zero under shade or under sun conditions. All creams except *Gly* and all lotions and creams showed higher PII when the cosmetics are applied under sun exposed skin (Table 3). Mann-Whitney *U* test indicated no significant difference between the creams applied once on intact and abraded skin (*P* = 0.71). For all creams, except Dove, the erythema appeared after 24 h and disappeared after 72 h. The untreated control group showed no erythema and edema. 

Regarding the lotions, *Bod* and Holly (*Hol*) caused PII of 0.06 on intact and PII of 0.1 to 0.17 on abraded skin. Magic Fruit showed the higher PII (0.47) among the lotions. PII of lotions significantly differed between applications on intact and abraded skin (*P* = 0.0021). However, no significant difference was observed between the PII under shade and under sun.

#### 3.3.2. Effect of Five Day Application of Cosmetics on Rabbit Skin

**Figure 1:**
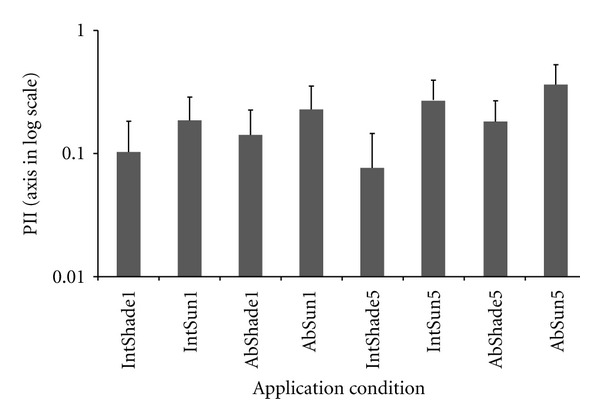
The average PII and standard deviation (error bar) of the cosmetics applied once and five consecutive days on a rabbit skin at different condition. IntShade1: applied once on intact skin under shade, IntSun1: applied once on intact skin under sun, AbShade1: applied once on abraded skin under shade, AbSun1: applied once on abraded skin under sun, IntShade5: applied five consecutive days on intact skin under shade, IntSun5: applied five consecutive days on intact skin under sun, AbShade5: applied five consecutive days on abraded skin under shade, AbSun5: applied five consecutive days on abraded skin under sun.

This test was conducted to determine any adverse skin reaction to creams when used continuously for five days with or without exposure to direct sunlight. The results showed that all creams tested caused a maximum PII of 0.17 without direct sunlight exposure and PII of 0.45 under direct sunlight. Dov could not show any sign of irritation when applied for five consecutive days except for abraded skin under sunlight. Among lotions, the application of *Sol* for five days resulted in the maximum PII of 0.7. In general maximum PII was observed for cosmetics applied on abraded skin under direct sunlight (Figure 1). Statistically significant difference has been observed between PII of abraded skin and intact skin (*P* = 0.012). Strong significant difference of PII was abserved for cosmetics applied under shade and under direct sunlight (*P* = 0.000).  Table 4 shows PII results of different cosmetics applied under different skin conditions.

**Figure 2:**
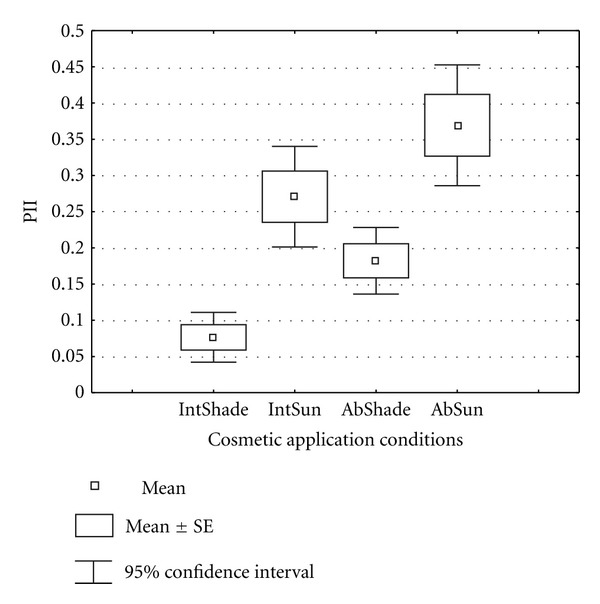
Box plot of primary irritation index (PII) of cosmetics applied under different environmental conditions on a rabbit skin. IntShad: intact skin under shade, IntSun: intact skin under sun, AbShade: abraded skin under shade, and AbSun: abraded skin under sun.

Post hoc analysis was made to compare the irritation index between cosmetics applied between intact and abraded skin under shade or sun-exposed conditions. Irritation index was higher for the cosmetics applied on abraded skin under sun exposed (AbSun) than the rest of the applications.  Figure 2 compares the four groups of cosmetic applications using PII of 95% confidence interval. PII resulted from application of cosmetics on intact skin under shade (IntShade) was significantly (*P* < 0.0001) lower than PII of cosmetics applied on both intact and abraded skin under sun exposed conditions. No significant difference (*P* = 0.08) was observed between PII of cosmetics applied on intact skin under shade (IntSun) and abraded skin under shade (IntShade).

According to the Mann-Whitney *U* Test, the PII calculated from cosmetics applied just once significantly differed from the application of five consecutive days (*P* value = 0.038). With similar statistical test, the PII calculated under shade significantly differed from the PII under sun (*P* = 0.000). Significant difference of PII also observed between intact and abraded skin (*P* = 0.006). Figure 1 explains the PII under different application conditions.

## 4. Discussion

Cosmetics and personal care products should be nontoxic, nonirritating, and must be safe for the consumers. The people use these products every day without knowing or being aware of the side effects of cosmetic products specifically in countries like Ethiopia where the literacy rate is very low. Therefore, in this study, an effort had been made to assess the possible *skin irritation* (dermal reaction), using rabbit as a model animal, that may result from the daily usage of these products in order to protect the consumers by educating the community and if necessary to call the attention of government about the need to regulate production and import of cosmetic products in the country. Our study has identified most cosmetics used by communities in Jimma town were unsafe for daily use. The absence strict safety regulation governing nonmedicated cosmetics by DACA most probably has lead to the presence of unsafe cosmetic products in shops. But some African countries like Tanzania have their own cosmetics directives similar to the European and Australian cosmetics directives [21].

Among the 14 cosmetic products selected, those who have no expiry date (*Col*, *Niv*, *Mag,* and *Kri*) could pose public health risk as consumers could use them at any time due to absence of shelf life. In developed countries, such as developed EC member countries, these products could not be sold for public use. The European Directive for Cosmetics (directive number 76/768/EEC) insists that products should have a clear label specifying the ingredient composition, country of manufacture, and expiry date of the products. 

Almost all of the products commonly used by consumers in Jimma town contain at least two ingredients that were prohibited from usage [36, 37]. Almost all of the examined cosmetics in our study contain paraben or parabenzoic acid (methyl paraben, propyl paraben) which is suspected of causing endocrine disruption and breast cancer [19, 38]. Darbre and Harvey (2008) in their review of resent studies have confirmed that parabens were found in urine after topical application of skin care products on healthy human skin [38]. Therefore, those products identified in our study may not be safe for human health. A similarly study conducted by EWG (2009) indicated that 833 name-brand sunscreens were found unsafe due to unhealthy ingredients [6].

Another risk identified in our study was the absence of list of ingredients on *Swe* and *Vic* which makes someone to suspect that there might be some toxic chemical component. These products do not meet the safety requirements for cosmetics and should not be allowed for public marketing. High level of mercury was found in cream cosmetics which have no product label [39]. Table 5 explains the ingredients and the possible health effects along with each cosmetic product.

Regarding the erythema and edema, creams showed the least PII compared to other cosmetics for both single- and five-day application. Among creams, Dov was found to be the safest which has no skin irritation except for five-day application on abraded skin under sun exposure. This also could indicate that the product may have no skin irritating chemicals in the products formula, and the manufacturing company could have been complied with the USFDA standards which urge that cosmetic products should be nonirritating. However, the harmful ingredient found in Dov (Table 5) has to be replaced by safest ones. 

The other cream cosmetics, *Col*, *Top,* and *Fai,* produce erythema and edema which were rated as PII of 0.06 to 0.18 for a single application. These products have negligible skin irritation and could be safe to be used by consumers as the PII ranges between 0–0.4 provided that the ingredients are also safe [34]. However, *Top* may not be safe on repeated application when used on abraded skin under sun exposure as the PII score was found to be greater than 0.4. 

 The PII of *Niv*, *Lux*, *Mag*, *Sol,* and *Bod* lotions was between 0.0 and 0.27 with single application under shade, indicating that the products have negligible skin irritancy and safe to use as topical application. However, based on single application under sun exposure, Magic Fruit may not be safe when used on abraded skin. On the basis of five-day application, *Lux*, *Sol*, *Kri*, and *Hol* may not be safe to human skin when used continuously under sun exposure, and the effect could be worse when used on abraded skin. This study has revealed that continuous application of those cosmetics on abraded skin under sun exposure could be dangerous for skin health. Hence individuals with abraded skin better not to use cosmetic products unless otherwise advised by their physicians. 

This study has identified that the cosmetics used may not be safe for topical application on human skin due to the ingredients found in them, absence of expiry date, and the skin irritation posed by them. Hence consumers have to be very curious and clearly know what they are using for their skin care. We have identified cosmetics containing chemical ingredients that could cause public health problem such as cancer, endocrine disruption, or low sperm count in males [36, 37]. Similarly, study done in Denmark has indicated that the prevalence of allergic contact sensitization to cosmetic haptens among younger adults in the general population was a public health problem [39].

Strategic followup and screening of body care products that are imported from abroad and produced in Ethiopia could help to partly safeguard consumers' health. At least each product should have labels, and the ingredients should not have a suspected effect on human health. In addition, public health personnel should work on awareness creation related to use of body care products. 

## 5. Conclusions

Cosmetics and personal care products should be nontoxic and should pose no risk under normal use. All The products tested for their skin reaction showed negligible skin irritancy but Dove had zero irritancy under the entire test conducted. But, there are prohibited ingredients in all of the products tested which made the products unsafe for the intended use. Some products have no appropriate label claims according to legislation set by USFDCA as well as the European cosmetic directives, and the products sold openly on the market are misbranded. This could expose consumers for public health risk. Therefore, consumers have to be aware about the side effects related to cosmetic products, and they should be informed to read the labels on the products' container. The consumers should avoid an attitude that cosmetic products are safe. Similarly, cosmetic industries, importers, distributors, and retailers should consider whether the product they sale to the consumer meets appropriate label requested by the DACA or not. Further study is suggested to know the chronic effect of those cosmetics in this study as well as laboratory analysis of the ingredients to identify mainly heavy metals.

## Figures and Tables

**Table 1:** United States Testing Company (USTC) erythematic and edema evaluation scale [31].

Erythema	Scale	Edema	Scale
No erythema	0	No edema	0
Very slight erythema (barely perceptible)	1	Very slight edema (barely perceptible)	1
Well-defined erythema	2	Slight edema (edges of area well defined by definite raising)	2
Moderate to severe erythema	3	Moderate edema (raised approximately 1 millimeter)	3
Severe erythema (beef redness) to eschar formation preventing grading of erythema	4	Severe edema (raised more than 1 millimeter and extending beyond the area exposed).	4

**Table 2:** Most commonly used cosmetic products by consumers and preference ranks given by shopkeepers in Jimma town, May, 2011. Cosmetics are listed using their abbreviations used in the result section.

Rank	Cosmetics	Country of origin	Percentage of consumer preference
1	*Dov *	India	86.7
2	*Gly *	Germany	73.3
3	*Bod *	S. Africa	66.7
4	*Col *	S. Africa	60
5	*Top *	India	53.3
6	*Fai *	India	53.3
7	*Niv *	Germany	53.3
8	*Sol *	Germany	53.3
9	*Lux *	S. Africa	46.7
10	*Mag *	China	40
11	*Hol *	Indonesia	40
12	*Vic *	Ethiopia	33.3
13	*Kri *	Ethiopia	20

Because of multiple responses, the sum of the percentages is above 100.

**Table 3:** Primary irritation index (PII) of creams, lotions and make-ups applied once on intact and abraded rabbit skin, calculated from combined erythema and edema ratings for three rabbits at three observation periods (24, 48, and 72 h), Jimma, 2011.

Cosmeti group	Brand	PII			
		Under shade		Under sun	
		Intac skin	Abrade skin	Intac skin	Abrade skin
	*Dov *	0	0	0	0
	*Gly *	0	0.06	0.12	0.18
Creams	*Col *	0.06	0.1	0.1	0.18
	*Top *	0.1	0.1	0.1	0.1
	*Fai *	0.1	0.1	0.1	0.1

	*Bod *	0	0.06	0.12	0.18
	*Lux *	0.06	0.17	0.28	0.39
	*Sol *	0.1	0.17	0.24	0.31
Lotions,	*Niv *	0.17	0.19	0.21	0.23
	*Mag *	0.17	0.27	0.37	0.47
	*Hol *	0.06	0.1	0.14	0.18
	*Kri *	0.17	0.2	0.23	0.26

Make-ups	*Swe *	0.28	0.3	0.32	0.34
	*Vic *	0.17	0.17	0.27	0.28
Control		0	0	0	0

**Table 4:** Primary irritation index (PII) of creams, lotions, and make-ups applied on rabbit skin for five consecutive days, under shade and under sun, Jimma town, 2011. The results were calculated from combined erythema and edema ratings for three rabbits at five observation days.

Cosmetic groups	Brands	PII			
		Under shade		Under sun	
		Intact skin	Abraded skin	Intact skin	Abraded skin
	*Dov *	0	0	0	0.1
	*Gly *	0	0.11	0.2	0.36
Creams	*Fai *	0.03	0.09	0.13	0.21
	*Col *	0.07	0.13	0.19	0.25
	*Top *	0.03	0.17	0.31	0.45

	*Niv *	0.13	0.21	0.29	0.37
	*Lux *	0	0.34	0.48	0.6
	*Mag *	0.07	0.13	0.19	0.25
Lotions	*Sol *	0.10	0.30	0.50	0.70
	*Bod *	0.20	0.21	0.21	0.23
	*Kri *	0.10	0.24	0.38	0.45
	*Hol *	0.03	0.17	0.31	0.45

Make-ups	*Vic *	0.14	0.22	0.31	0.4
	*Swe *	0.17	0.23	0.29	0.35
Control		0	0	0	0

**Table 5:** Prohibited ingredients identified due to their health effects among the cosmetics commonly used in Jimma town [36, 37].

Products	Prohibited ingredients	Possible health risk
All creams and *Mag *	Sodium methylparabens and propylparabens*	Can have endocrine-disrupting action, breast cancer
*Dov*, *Gly*, *Fai*, and *Lux *	Linalool	Causes CNS disorder
*Dov, Gly, Lux *	Limonene	Carcinogenic, it is an irritant and sensitizer
*Dov, Fai, Lux, Mag *	Disodium EDTA	Contains dangerous levels of ethylene oxide and or dixane, both potent toxins
*Top, Bod *	Sodium lauryl sulphate	Irritant
*Dov, Col, Hol *	Triethanolamine (TEA)	Induces nitrosamines, which are potent carcinogens

*propylparabens are banned for under 3 children in Denmark.
